# CHA_2_DS_2_-VASc score predicts exercise intolerance in young and middle-aged male patients with asymptomatic atrial fibrillation

**DOI:** 10.1038/s41598-018-36185-7

**Published:** 2018-12-21

**Authors:** Jeong-Eun Yi, Young Soo Lee, Eue-Keun Choi, Myung-Jin Cha, Tae-Hoon Kim, Jin-Kyu Park, Jung-Myung Lee, Ki-Woon Kang, Jaemin Shim, Jae-Sun Uhm, Jun Kim, Changsoo Kim, Jin-Bae Kim, Hyung Wook Park, Boyoung Joung, Junbeom Park

**Affiliations:** 1 Department of Cardiology, College of Medicine, Ewha Womans University School of Medicine, Seoul, Republic of Korea; 2 Division of Cardiology, Catholic University of Daegu, Daegu, Republic of Korea; 3 Department of Internal Medicine, Seoul National University Hospital, Seoul, Republic of Korea; 4 Division of Cardiology, Department of Internal Medicine, Yonsei University College of Medicine, Seoul, Republic of Korea; 5 Division of Cardiology, Hanyang University Medical College, Seoul, Republic of Korea; 6 Division of Cardiology, Kyung Hee University Medical College, Seoul, Republic of Korea; 7 Division of Cardiology, Eulji University Hospital, Daejeon, Republic of Korea; 8 Division of Cardiology, Korea University Anam Hospital, Seoul, Republic of Korea; 9 Department of Internal Medicine, University of Ulsan College of Medicine, Seoul, Republic of Korea; 10 Department of Preventive Medicine, Yonsei University College of Medicine, Seoul, Republic of Korea; 11 Department of Cardiovascular Medicine, Chonnam National University Medical School, Gwangju, Republic of Korea

## Abstract

Exercise intolerance among the clinical symptoms in patients with atrial fibrillation (AF) has usually been masked by their adjusted life style. We sought to assess the role of CHA_2_DS_2_-VASc score to predict exercise intolerance in asymptomatic AF patients, and further examine whether the relationship differs by age and gender. Among the 6,275 participants of the prospective Korean registry of the Comparison study of Drugs for symptom control and complication prevention of Atrial Fibrillation (CODE-AF), 1,080 AF patients who underwent exercise treadmill testing were studied. Exercise intolerance was defined as a peak exercise capacity of 7 metabolic equivalents (METs) or less, and the patients were divided into two groups for the analysis: ≤7 METs (n = 131) and >7 METs (n = 949). Patients with exercise intolerance had a significantly higher CHA_2_DS_2_-VASc score than those without (3.1 ± 1.3 vs. 2.0 ± 1.5, p < 0.0001). In the multivariate analysis, a higher CHA_2_DS_2_-VASc score (OR 1.54, 95% CI 1.31–1.81, p < 0.0001), corrected QT interval (OR 1.01, 95% CI 1.00–1.02, p = 0.026), and increased left atrial volume index (OR 1.02, 95% CI 1.01–1.03, p = 0.001) were found to be independent predictors of exercise intolerance. The impact of the CHA_2_DS_2_-VASc score on exercise intolerance was significant only in male patients aged <65 years (OR 3.30, 95% CI 1.76–6.19, p < 0.0001). The CHA_2_DS_2_-VASc score may be a feasible risk assessment tool to predict exercise intolerance, especially in young and middle-aged male patients with asymptomatic AF.

## Introduction

Atrial fibrillation (AF) is the most common sustained cardiac arrhythmia, with a prevalence of 1–2% in the general population^1,2^. Many patients with AF experience various degrees of impaired exercise tolerance, which is associated with an increased morbidity and mortality, and poor quality of life^3–5^. However, some patients with AF are asymptomatic^6^ and their adjusted life style may also mask the exercise intolerance. Since asymptomatic AF patients are more likely to miss out on the appropriate treatment options, their prognosis is worse than that of symptomatic patients^7–9^. Therefore, early detection and treatment of exercise intolerance in these patients have important clinical implications. Exercise Treadmill testing (TMT) may be a useful tool to estimate exercise capacity in AF patients, however; it cannot be routinely performed in patients with mental or physical impairment, or conversely, in apparently healthy subjects without symptom^10^.

The CHA_2_DS_2_-VASc score (Congestive HF, hypertension, age ≥75 years [doubled], type 2 diabetes, previous stroke or transient ischemic attack (TIA) [doubled], vascular disease, age 65 to 74 years, and sex category) has been originally recommended for the stroke risk stratification in AF^1^. Recently, its usefulness as a risk assessment tool for adverse clinical outcomes other than thromboembolic events has also been explored beyond the AF field^11–13^. The aim of our study was to investigate the association between CHA_2_DS_2_-VASc score and exercise capacity in AF patients. In this study, we tested the hypothesis that the CHA_2_DS_2_-VASc score could predict exercise intolerance in asymptomatic AF patients. And we further examined whether the relationship differs by age and gender, because age and gender were very important risk factors which influence on exercise intolerance.

## Methods

### Study population

The COmparison study of Drugs for symptom control and complication prEvention of AF (CODE-AF) is a prospective, multicenter, ongoing observational study conducted in patients aged >18 years with AF at 10 tertiary hospitals in South Korea. The primary aim of the CODE-AF registry is to compare the outcomes according to the different medical treatment strategies such as anticoagulation, rate control, and rhythm control. The secondary aim of the study is to describe the clinical epidemiology of AF and to evaluate the diagnostic and therapeutic processes applied to patients with AF and their clinical outcomes. The registry was funded and designed by the Korea Ministry of Health & Welfare (HI15C1200), which provided national coordinators. And it was coordinated by the Korea Heart Rhythm Society, which provides support to related committees and participating centers. Data are entered into a common electronic database that limits inconsistencies and errors and provides online help for key variables. Each center has access not only to its own data, but also to data from all other participating centers. AF-related symptoms included palpitations, tachycardia, syncope or presyncope, dyspnea, orthopnea, shortness of breath on exertion, chest pain, fatigue, or malaise, which were assessed by a self-reported questionnaire and classified into three grades based on the EHRA symptom scale. The details of the study design has been reported previously^14^. This study was based on the first released database for enrolled patients between June 2016 and April 2017, who were >18 years with non-valvular AF, attended an outpatient clinic, and were hospitalized on the same day for AF. All patients were scheduled for clinically follow-up every 6 months, either through personal interview or telephone contact. This study was conducted in accordance with the Declaration of Helsinki and the relevant guidelines and regulations. The study protocol was approved by the research Ethics Committee of Ewha Womans University Mokdong Hospital (No. 216-02-056), and all patients gave their written informed consent prior to enrollment. This study was registered in the ClinicalTrials.gov (NCT02786095).

Among the 6,275 participants of the CODE-AF, 1,872 asymptomatic AF patients who underwent exercise TMT as a screening exam were selected. From those, 792 patients were excluded for the following reasons: a history of congestive HF or myocardial infarction (n = 190), structural or valvular heart disease (n = 142), or radiofrequency catheter ablation of AF (n = 417); presence of an intracardiac device such as a pacemaker or implantable cardioverter defibrillator (n = 89); left ventricular (LV) systolic dysfunction (ejection fraction [EF] <50%) (n = 105); the presence of stenosis (>50%) on coronary computed tomography (CT) or coronary angiography (n = 121); or missing data (n = 77). Finally, a total of 1,080 consecutive patients were eligible for our analysis. The CHA_2_DS_2_-VASc score was calculated for each patient based on their demographic and clinical information at the time of enrollment, and categorized into three groups (0–1 points, 2–3 points, and ≥4 points).

### Exercise protocol

All patients underwent maximal, symptom-limited exercise TMT with electrographic monitoring using the Bruce or Naughton protocol. ST-segment depression was defined as a ≥1 mm horizontal or down-sloping at 80msec from the J point for 3 consecutive beats^10^. The exercise capacity was measured in peak metabolic equivalents (METs) estimated from the basis of the exercise speed and grade using the standardized equations. Exercise intolerance was defined as a peak exercise capacity of ≤7 METs^15^.

### Statistical analysis

The continuous variables are presented as the mean ± SD, whereas categorical variables are presented as counts and percentages. Comparisons of the variables across the groups were performed using a Student *t* test or a one-way ANOVA combined with a Bonferroni post hoc analysis for continuous variables and Chi-square (χ^2^) or Fisher’s exact test for categorical variables, as appropriate. A multiple logistic regression analysis was performed to determine the independent predictors of exercise intolerance using covariates identified as significant in the univariate analysis or previously known to be important variables. A receiver operating characteristic (ROC) curve was constructed to assess the predictive ability of the CHA_2_DS_2_-VASc score for exercise intolerance. All statistical analyses were performed using the SPSS version 21.0 software package (IBM SPSS, New York, USA). A *P* < 0.05 was considered to be statistically significant.

## Results

### Patient demographic characteristics

The 1,080 patients had a mean age of 65 ± 11 years and consisted of 769 (71.2%) men. The mean CHA_2_DS_2_-VASc score was 2.1 ± 1.5, demonstrating a low CHA_2_DS_2_-VASc score (0–1) in 407 (37.7%) patients, intermediate score (2–3) in 492 (45.6%), and high score (≥4) in 181 (16.8%). The mean peak exercise capacity was 10.5 ± 2.7 METs, and the patients were divided into two groups according to the exercise capacity: ≤7 METs (n = 131) and >7 METs (n = 949).

The baseline characteristics are presented in Table 1. The patients with exercise intolerance were older, and had a higher prevalence of chronic kidney disease and higher body mass index (BMI). The prevalence of the components constituting the CHA_2_DS_2_-VASc score, including hypertension, diabetes mellitus, an age ≥75, an age 65–74, and a female gender were higher in the patients with exercise intolerance. However, there were no significant differences in the proportion of subjects with a stroke or TIA, peripheral artery disease, dyslipidemia, or smoking between the two groups. Persistent AF was more frequently found in patients with exercise intolerance, but the duration of AF was similar between the two groups. Angiotensin converting enzyme inhibitors (ACEis) or angiotensin receptor blockers (ARBs) and calcium channel blockers (CCBs) were more commonly used in patients with exercise intolerance, whereas the use of beta-blockers, digoxin, and antiarrhythmic drugs were similar between the two groups.

**Table 1:** Demographic characteristics.

Variables	Total (n = 1,080)	≤7 METs (n = 131)	>7 METs (n = 949)	P value*
Age (years)	65 ± 11	71 ± 8	64 ± 11	<0.0001
Age <65	509 (47.1)	23 (17.6)	486 (51.2)	<0.0001
Age 65–74	391 (36.2)	64 (48.9)	327 (34.5)	0.001
Age ≥75	180 (16.7)	44 (33.6)	136 (14.3)	<0.0001
Male	769 (71.2)	70 (53.4)	699 (73.7)	<0.0001
Body mass index (kg/m^2^)	24.9 ± 3.2	25.5 ± 3.7	24.8 ± 3.1	0.048
Duration of AF ( ≥ 3 months)	932 (86.3)	114 (87.0)	818 (86.2)	0.893
Type of AF				
Paroxysmal	817 (75.6)	89 (67.9)	728 (76.7)	0.028
Persistent	234 (21.7)	40 (30.5)	194 (20.4)	0.009
Permanent	29 (2.7)	2 (1.5)	27 (2.8)	0.566
Diabetes mellitus	263 (24.4)	48 (36.6)	215 (22.7)	<0.0001
Hypertension	747 (69.4)	114 (87.0)	633 (66.9)	<0.0001
Stroke/TIA	111 (10.3)	97 (10.2)	14 (10.7)	0.892
Peripheral artery disease	22 (22.0)	5 (3.8)	17 (1.8)	0.174
Dyslipidemia	437 (40.7)	54 (41.5)	383 (40.5)	0.826
Chronic kidney disease	100 (9.3)	20 (15.3)	80 (8.4)	0.011
Smoking	405 (37.5)	41 (31.3)	364 (38.4)	0.118
Medications				
ACEi or ARB	381 (35.3)	65 (49.6)	316 (33.3)	<0.0001
Beta-blocker	529 (49.0)	72 (55.0)	457 (48.2)	0.144
Calcium channel blocker	339 (31.4)	59 (45.0)	280 (29.5)	<0.0001
Digoxin	35 (3.2)	7 (5.3)	28 (3.0)	0.181
Antiarrhythmic drug	624 (57.8)	70 (53.4)	554 (58.4)	0.283

*P value: ≤7 METs vs. >7 METs.

Values are presented as the mean ± SD or n (%).

Abbreviations: MET = metabolic equivalent; TIA = transient ischemic attack; AF = atrial fibrillation; ACEi = angiotensin converting enzyme inhibitor; ARB = angiotensin receptor blocker.

### Difference in the clinical characteristics of the patients with and without exercise intolerance

**Figure 1:**
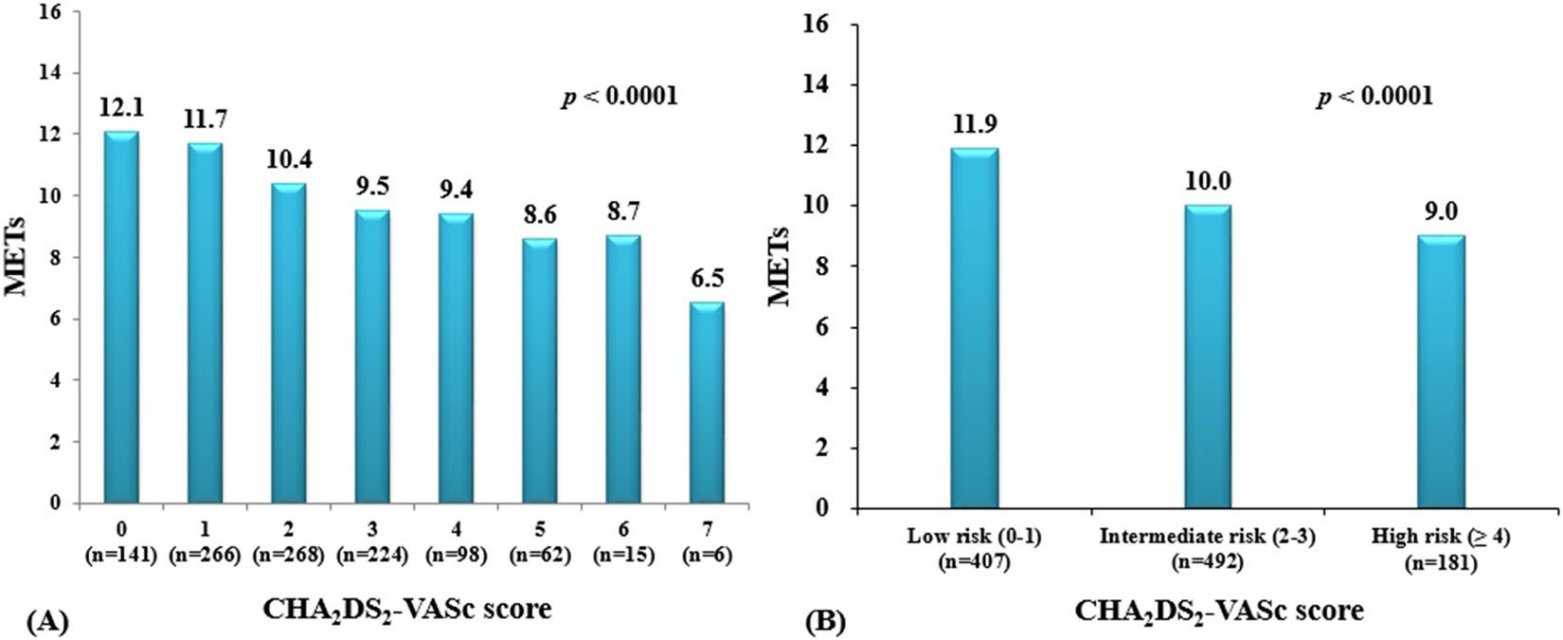
(**A**) Changes in the peak exercise capacity according to the CHA_2_DS_2_-VASc scores (n = 1,080). (**B**) Categorical analysis for changes in peak exercise capacity among each risk group according to the CHA_2_DS_2_-VASc scores (low vs. intermediate risk group, *p* < 0.0001; intermediate vs. high risk group, *p* < 0.0001; low vs. high risk group, *p* < 0.0001).

Patients with exercise intolerance had a significantly higher CHA_2_DS_2_-VASc score (3.1 ± 1.3 vs. 2.0 ± 1.5, *P* < 0.0001) and larger proportion of intermediate or high-risk categories (Table 2). The peak exercise capacity according to the CHA_2_DS_2_-VASc score is shown in Fig. 1. As the CHA_2_DS_2_-VASc scores increased from 0 to 7, the peak exercise capacity significantly decreased from 12.1 to 6.5 METs (*P* < 0.0001) (Fig. 1A), and the categorical analysis also showed a significant difference among each risk group according to the CHA_2_DS_2_-VASc score (*P < *0.0001) (Fig. 1B).

**Table 2:** Electrocardiography, Echocardiography, and Exercise treadmill test variables.

Variables	Total (n = 1,080)	≤7 METs (n = 131)	>7 METs (n = 949)	P value*
CHA_2_DS_2_-VASc score	2.1 ± 1.5	3.1 ± 1.3	2.0 ± 1.5	<0.0001
Low (0–1)	407 (37.7)	8 (6.1)	399 (42.0)	<0.0001
Intermediate (2–3)	492 (45.6)	81 (61.8)	411 (43.3)	<0.0001
High (≥4)	181 (16.8)	42 (32.1)	139 (14.6)	<0.0001
Electrocardiography				
QRS duration (ms)	99 ± 19	97 ± 18	100 ± 19	0.147
QTc interval (ms)	435 ± 32	443 ± 30	434 ± 32	0.001
Echocardiography				
LVEF (%)	64.3 ± 6.2	64.5 ± 6.1	64.3 ± 6.2	0.687
E/E’	10.5 ± 4.1	12.3 ± 4.4	10.3 ± 4.0	<0.0001
LAVI (ml/m^2^)	39.5 ± 19.4	50.1 ± 28.7	38.0 ± 17.2	<0.0001
Exercise treadmill test				
Resting SBP (mmHg)	122 ± 14	124 ± 14	122 ± 14	0.079
Resting DBP (mmHg)	76 ± 11	73 ± 12	76 ± 11	0.003
Resting HR (bpm)	73 ± 15	74 ± 16	73 ± 14	0.206
Peak exercise HR (bpm)	162 ± 35	137 ± 33	166 ± 34	<0.0001
Achieved ≥85% of MAPHR	875 (81.0)	76 (58.0)	799 (84.2)	<0.0001
Peak exercise capacity (MET)	10.5 ± 2.7	5.7 ± 1.3	11.2 ± 2.0	<0.0001
Inducible ST depression	134 (12.4)	20 (15.3)	114 (12.0)	0.290

*P value: ≤7 METs vs. >7 METs.

Values are presented as the mean ± SD or n (%).

Abbreviations: MET = metabolic equivalent; LVEF = left ventricular ejection fraction; LAVI = left atrial volume index; SBP = systolic blood pressure; DBP = diastolic blood pressure; HR = heart rate; MAPHR = maximum age predicted heart rate.

Patients with exercise intolerance had a significantly increased QTc interval and had higher E/E’ and left atrial volume index (LAVI) than those achieving a peak of more than 7 METs. The resting diastolic blood pressure (DBP) and peak exercise heart rate (HR) were significantly lower in the patients with exercise intolerance than in those without, as was the proportion of patients achieving ≥85% of their maximum age-predicted heart rate (MAPHR). There were no significant differences in the prevalence of exercise-induced ST-segment depression between the groups.

### Predictors of exercise intolerance in the asymptomatic AF patients

Logistic regression analyses for the association of the clinical variables to exercise intolerance are presented in Table 3. In the univariate analyses, the CHA_2_DS_2_-VASc score, BMI, chronic kidney disease, persistent AF, QTc interval, E/E’, LAVI, and resting DBP were related to exercise intolerance. After an adjustment for the significant covariates, a high CHA_2_DS_2_-VASc score (adjusted odds ratio [OR] 1.54, 95% confidence interval [CI] 1.31–1.81, *P* < 0.0001), prolonged QTc interval (adjusted OR 1.01, 95% CI 1.00–1.02, *P* = 0.026), and enlarged LAVI (adjusted OR 1.02, 95% CI 1.01–1.03, *P* = 0.001) remained as independent determinants of exercise intolerance.

**Table 3:** Univariate and multivariate analysis for exercise intolerance.

Variables	Univariate analysis			Multivariate analysis		
	OR	95% CI	P value	OR	95% CI	P value
CHA_2_DS_2_-VASc score*	1.60	1.41–1.80	<0.0001	1.54	1.31–1.81	<0.0001
BMI (kg/m^2^)	1.06	1.01–1.12	0.025	1.05	0.99–1.12	0.115
Smoking	0.73	0.50–1.08	0.119	1.34	0.83–2.16	0.229
Chronic kidney disease	1.96	1.15–3.32	0.013	0.97	0.50–1.89	0.924
Persistent AF**	1.69	1.13–2.53	0.011	1.25	0.74–2.13	0.407
Permanent AF**	0.61	0.14–2.59	0.499	0.79	0.09–6.76	0.832
QTc interval (ms)	1.01	1.00–1.02	0.001	1.01	1.00–1.02	0.026
LVEF (%)	1.01	0.98–1.04	0.542	1.01	0.97–1.05	0.659
E/E’	1.10	1.05–1.15	<0.0001	1.01	0.96–1.07	0.656
LAVI (ml/m^2^)	1.02	1.02–1.03	<0.0001	1.02	1.01–1.03	0.001
Resting DBP (mmHg)	0.97	0.96–0.99	0.003	0.98	0.96–1.00	0.054
Resting HR (bpm)	1.01	0.99–1.02	0.206	1.01	0.99–1.02	0.259
Use of beta-blocker	1.31	0.91–1.90	0.145	1.14	0.73–1.78	0.572

*Excluding congestive heart failure component, **versus paroxysmal AF.

Abbreviations: OR = odds ratio; CI = confidence interval; BMI = body mass index; AF = atrial fibrillation; LVEF = left ventricular ejection fraction; LAVI = left atrial volume index; DBP = diastolic blood pressure; HR = heart rate.

**Figure 2:**
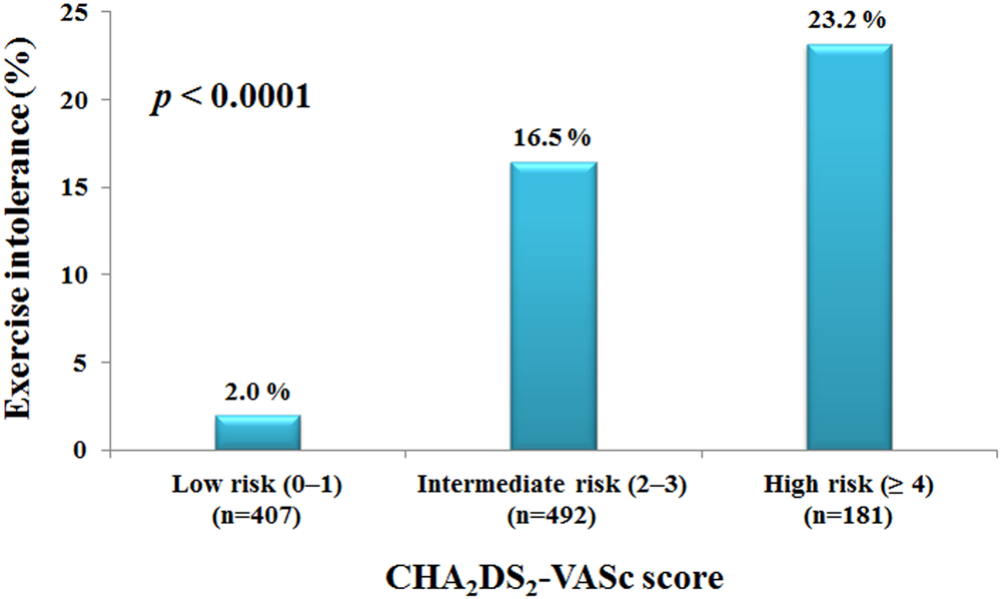
Prevalence of exercise intolerance in patients with low, intermediate, and high risk categories of CHA_2_DS_2_-VASc scores.

Patients with a CHA_2_DS_2_-VASc score of 2–3 (16.5%) and CHA_2_DS_2_-VASc score of ≥4 (23.2%) were more likely to experience an impaired exercise capacity compared to those with a CHA_2_DS_2_-VASc score of 0–1 (2.0%) (*P* < 0.0001) (Fig. 2). Further, the association between CHA_2_DS_2_-VASc risk stratification and exercise intolerance was significant even after adjusting for the other confounding factors (Supplementary Table 1). In detail, the predictive value of the individual components of the CHA_2_DS_2_-VASc score were assessed, and we found that hypertension, diabetes mellitus, an age ≥75, an age 65 to 74, and a female gender were associated with exercise intolerance (Table 4).

**Table 4:** Predictive value of the individual components in the CHA_2_DS_2_-VASc score.

Variables	Univariate analysis			Multivariate analysis		
	OR	95% CI	P value	OR	95% CI	P value
Congestive heart failure	N/A					
Hypertension	3.16	1.89–5.30	<0.0001	2.28	1.31–3.96	0.004
Diabetes mellitus	1.97	1.34–2.90	0.001	1.61	1.07–2.42	0.024
Age ≥75 years*	6.84	3.99–11.7	<0.0001	5.03	2.87–8.81	<0.0001
Age 65–74 years*	4.14	2.52–6.80	<0.0001	3.34	2.01–5.56	<0.0001
Stroke/TIA	1.06	0.59–1.92	0.843	0.69	0.37–1.29	0.242
Peripheral artery disease	2.18	0.79–6.00	0.133	2.40	0.82–7.01	0.110
Female	2.44	1.68–3.54	<0.0001	2.13	1.44–3.15	<0.0001

*Versus age <65 years.

Abbreviations: OR = odds ratio; CI = confidence interval; N/A = not applicable; TIA = transient ischemic attack.

### Detection of exercise intolerance in young male AF patients

**Figure 3:**
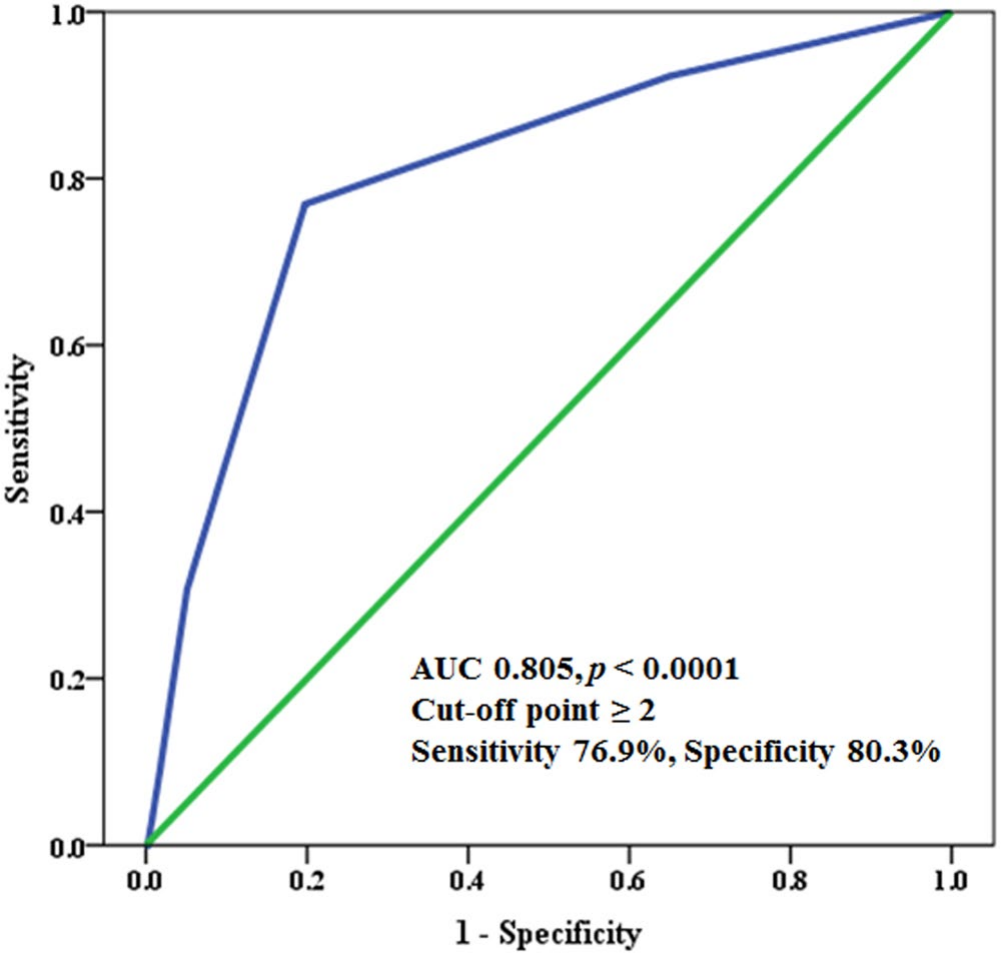
ROC curve analysis of the association between the CHA_2_DS_2_-VASc score and exercise intolerance in male patients aged <65 (n = 403). ROC = receiver operating characteristic.

Finally, we performed subgroup analyses to evaluate whether there were age or gender-specific differences between the CHA_2_DS_2_-VASc score and exercise intolerance (Table 5). Interestingly, a significant association between the CHA_2_DS_2_-VASc score and exercise intolerance was found just in male patients aged <65 years (adjusted OR 3.15, 95% CI 1.84–5.38, *P* < 0.0001), whereas it was not in male patients aged ≥65 years and female patients. In the ROC analysis conducted among the male patients aged <65 years, a CHA_2_DS_2_-VASc score of ≥2 was identified as the optimal cut-off value for predicting exercise intolerance (AUC 0.805, 95% CI 0.68–0.93, *P* < 0.0001) with a sensitivity of 76.9% and specificity of 80.3% (Fig. 3).

**Table 5:** Subgroup analyses stratified by age and gender.

	Adjusted OR	95% CI	P value
<65 years*			
Overall (n = 509)	3.15	1.84–5.38	<0.0001
Male (n = 403)	3.30	1.76–6.19	<0.0001
Female (n = 106)	2.28	0.78–6.70	0.134
≥65 years**			
Overall (n = 571)	1.17	0.98–1.41	0.091
Male (n = 366)	1.05	0.79–1.41	0.730
Female (n = 205)	1.19	0.88–1.63	0.265

*Adjusted for the BMI, smoking, AF type, LVEF, E/E’, LAVI, resting HR, and the use of beta-blockers; **Adjusted for the BMI, smoking, AF type, QTc interval, LVEF, E/E’, LAVI, resting DBP, resting HR, and the use of beta-blockers.

Abbreviations: OR = odds ratio; CI = confidence interval; BMI = body mass index; AF = atrial fibrillation, QTc = corrected QT; LVEF = left ventricular ejection fraction; LAVI = left atrial volume index; DBP = diastolic blood pressure; HR = heart rate.

## Discussion

Although a reduced exercise capacity is commonly found in AF patients, the exact mechanisms remain uncertain. Recently, Haskiah F *et al*. documented the association between the CHA_2_DS_2_-VASc score and improvement in the functional capacity, however, their study population consisted of acute coronary syndrome patients referred for cardiac rehabilitation^16^. Approximately one third of patients with AF have no obvious AF-related symptoms and no noticeable deterioration in quality of life^6^. Exercise intolerance in these patients is often diagnosed later and, thus, results in worse outcomes compared with symptomatic patients^7–9^. We examined for the first time the clinical application of the CHA_2_DS_2_-VASc score for assessing the exercise capacity in asymptomatic AF patients. Further, our results showed that a high CHA_2_DS_2_-VASc score may predict exercise intolerance, especially in young and middle-aged male patients (<65 years).

Exercise intolerance in patients with normal LV systolic function is associated with diastolic heart failure (DHF), also described as HF with preserved EF (HFpEF)^17^. Epidemiological studies have documented that AF itself causes the LA and LV remodeling, which, in turn, contributes to diastolic dysfunction and HFpEF^18–21^. However, HFpEF is a challenging diagnosis since it requires typical symptoms or signs of HF and evidence of diastolic dysfunction, but a normal LV systolic function^22^. Exercise hemodynamics, especially in stable outpatients with normal EF is largely determined by LV diastolic filling during exercise. For this reason, exercise testing has been used not only to identify masked HFpEF, but also to predict the prognosis in patients with HFpEF^23,24^. Our results have important implications in that the CHA_2_DS_2_-VASc score may be used as a feasible risk assessment tool for the early detection of HFpEF in apparently asymptomatic AF patients. However, in our study, patients with exercise intolerance were more likely to be older and females. Moreover, among the CHA_2_DS_2_-VASc components, age and female were powerful determinants of exercise intolerance. Although old age and female gender are also risk factors for HFpEF^22^, the possibility of age- or sex-related declines in functional capacity cannot be excluded^25^. Actually, we found that a high CHA_2_DS_2_-VASc score was independently associated with exercise intolerance only in male patients younger than 65 years. Considering that CHA_2_DS_2_-VASc score among them is determined only by cardiovascular risk factors except for the age and gender, this finding may further support the clinical role of CHA_2_DS_2_-VASc score in predicting HFpEF.

Additionally, the QTc interval was an independent predictor of exercise intolerance in this study. The QTc interval has been reported as an electrocardiographic parameter of LV diastolic dysfunction, and a prolonged QTc interval is known to be a strong predictor of death due to worsening HF^26^. Unlike prior studies, our study did not show a significant relationship between the resting HR and exercise capacity. However, most data have been derived from studies in patients with sinus rhythm, and the prognostic impact of rate control in patients with AF is still controversial^27,28^. Moreover, the type of AF was not associated with the exercise capacity in the present study, which was inconsistent with the previously published report showing a higher prevalence of HF in patients with persistent or permanent AF than in those with paroxysmal AF^29^. The detrimental impact of AF on the exercise hemodynamics is well known, but most studies have been conducted in patients with HF or have not focused on the type of AF^30–32^. It is speculated that the presence of AF, rather than the type of AF may be a more important factor for determining the exercise capacity in asymptomatic AF patients with a normal LV global systolic function. We also found that an increased LAVI was associated with exercise intolerance, which is consistent finding with the previous reports^33^. On the other hand, E/E’ was not a significant factor related to exercise intolerance in our study. Some studies have been concerned about the limited clinical value of the E/E’ in predicting LV filling pressure in patients with normal LVEF, as well as in those with AF^34^. LAVI seems to be a more reliable echocardiographic parameter for the diagnosis of HFpEF as compared to the E/E’ in patients with AF.

### Study limitations

This study had several limitations. First, our study included AF patients who underwent TMT as a screening exam. However, TMT was not routinely performed at all hospitals and thus, it was likely that patients with relatively more CV risk factors were referred for TMT at tertiary hospitals. Second, we arbitrarily defined exercise intolerance as ≤7 METs. Although various MET values have been used to predict adverse prognosis, there has been no widely accepted abnormal value for asymptomatic subjects^15^. Third, CODE-AF is an observational and multicenter registry conducted in patients with AF, so we lacked detailed information on the use of diuretics or whether medications were withheld before the exercise stress test. Moreover, the N-terminal pro B-type natriuretic peptide, the gold standard biomarker of HF, was not measured in the patients without symptoms or signs of HF. Finally, cardiopulmonary exercise testing that provides more comprehensive data on the exercise physiology than traditional exercise stress test was not available^10^.

## Conclusion

A higher CHA_2_DS_2_-VASc score was independently associated with a lower exercise capacity. Our findings suggested that the CHA_2_DS_2_-VASc score may be used to predict exercise intolerance, especially in relatively young and middle-aged male patients with asymptomatic AF. Further prospective studies with a long-term follow-up might be needed to confirm the prognostic significance of this score for identifying AF patients at a higher risk of HF.

## Electronic supplementary material

Supplementary Table 1
