# Supplementary material for: CHA2DS2-VASc score predicts exercise intolerance in young and middle-aged male patients with asymptomatic atrial fibrillation
Source: Sci Rep. 2018 Dec 21;8:18039. doi: 10.1038/s41598-018-36185-7 (PMC6303333; doi:10.1038/s41598-018-36185-7)
Supplement: Supplementary file 1 — Supplementary Table 1 [file 41598_2018_36185_MOESM1_ESM.pdf]

CHA_2DS_2-VASc score predicts exercise intolerance in young and middle-aged male patients with asymptomatic atrial fibrillation.

Jeong-Eun Yi[1], Young Soo Lee[2], Eue-Keun Choi[3], Myung-Jin Cha[3], Tae-Hoon Kim[4], Jin-Kyu Park[5], Jung-Myung Lee[6], Ki-Woon Kang[7], Jaemin Shim[8], Jae-Sun Uhm[4], Jun Kim[9], Changsoo Kim[10], Jin-Bae Kim[6], Hyung Wook Park[11], Boyoung Joung[4], Junbeom Park[1]

[1]Department of Cardiology, College of Medicine, Ewha Womans University School of Medicine, Seoul, Republic of Korea. [2]Division of Cardiology, Catholic University of Daegu, Daegu, Republic of Korea. [3]Department of Internal Medicine, Seoul National University Hospital, Seoul, Republic of Korea. [4]Division of Cardiology, Department of Internal Medicine, Yonsei University College of Medicine, Seoul, Republic of Korea. [5]Division of Cardiology, Hanyang University Medical College, Seoul, Republic of Korea. [6]Division of Cardiology, Kyung Hee University Medical College, Seoul, Republic of Korea. [7]Division of Cardiology, Eulji University Hospital, Daejeon, Republic of Korea. [8]Division of Cardiology, Korea University Anam Hospital, Seoul, Republic of Korea. [9]Department of Internal Medicine, University of Ulsan College of Medicine, Seoul, Republic of Korea. [10]Department of Preventive Medicine, Yonsei University College of Medicine, Seoul, Republic of Korea. [11]Department of Cardiovascular Medicine, Chonnam National University Medical School, Gwangju, Republic of Korea.

Dr. Jeong-Eun Yi and Dr. Young Soo Lee contributed equally to this work

Dr. Junbeom Park and Dr. Boyoung Joung are joint senior authors

Author Contributions

Yi JE: Conception and design of the study, or acquisition of data, or analysis and interpretation of data, Drafting the article or revising it critically for important intellectual content; **Lee YS:** Conception and design of the study, or acquisition of data, or analysis and interpretation of data; **Choi EK:** Conception and design of the study, or acquisition of data, or analysis and interpretation of data; **Cha MJ:** Conception and design of the study, or acquisition of data, or analysis and interpretation of data; **Kim TH:** Conception and design of the study, or acquisition of data, or analysis and interpretation of data; **Park JK:** Conception and design of the study, or acquisition of data, or analysis and interpretation of data; **Lee JM:** Conception and design of the study, or acquisition of data, or analysis and interpretation of data; **Kang KW:** Conception and design of the study, or acquisition of data, or analysis and interpretation of data; **Shim J:** Conception and design of the study, or acquisition of data, or analysis and interpretation of data; **Uhm JS:** Conception and design of the study, or acquisition of data, or analysis and interpretation of data; **Kim J:** Conception and design of the study, or acquisition of data, or analysis and interpretation of data; **Kim C:** Conception and design of the study, or acquisition of data, or analysis and interpretation of data; **Kim JB:** Conception and design of the study, or acquisition of data, or analysis and interpretation of data; **Park HW:** Conception and design of the study, or acquisition of data, or analysis and interpretation of data; **Joung B:** Conception and design of the study, or acquisition of data, or analysis and interpretation of data, Drafting the article or revising it critically for important intellectual content; **Park J:** Conception and design of the study, or acquisition of data, or analysis and interpretation of data, Drafting the article or revising it critically for important intellectual content, Final approval of the version to be submitted.

Conflict of interest

There is no conflict of interest

Correspondence to:

Boyoung Joung, MD, PhD

Professor of Medicine, Cardiology Division,

Yonsei Cardiovascular Hospital, Yonsei University College of Medicine,

250 Seungsanno, Seodaemun-gu, Seoul 120-752

Phone: +82-2-2228-8460

Fax: +82-2-393-2041

Email: cby6908@yuhs.ac

Junbeom Park, MD, PhD

College of Medicine, Ewha Womans University

1071, Annyangcheon-ro, Yangcheon-gu, Seoul

Phone: +82-2-2650-5826

Email: parkjb@ewha.ac.kr

Supplementary Table 1 Independent association between CHA_2DS_2-VASc risk stratification and exercise intolerance

Variables	Univariate analysis			Multivariate analysis*		
	OR	95% CI	P value	OR	95% CI	P value
CHA_2DS_2-VASc group						
Low (0-1)	Reference			Reference		
Intermediate (2-3)	9.86	4.70–20.64	<0.0001	7.53	3.14–18.07	<0.0001
High (≥ 4)	15.07	6.91–32.89	<0.0001	12.36	4.81–31.76	<0.0001

*Adjusted for the BMI, smoking, chronic kidney disease, type of AF, QTc interval, LVEF, E/E’, LAVI, resting DBP, resting HR, and use of beta-blockers.

Abbreviations: OR = odds ratio; CI = confidence interval; BMI = body mass index; AF = atrial fibrillation; LVEF = left ventricular ejection fraction; LAVI = left atrial volume index; DBP = diastolic blood pressure; HR = heart rate.
